# Identification of a biosynthetic gene cluster for the polyene macrolactam sceliphrolactam in a *Streptomyces* strain isolated from mangrove sediment

**DOI:** 10.1038/s41598-018-20018-8

**Published:** 2018-01-25

**Authors:** Zhen Jie Low, Li Mei Pang, Yichen Ding, Qing Wei Cheang, Kim Le Mai Hoang, Hoa Thi Tran, Jinming Li, Xue-Wei Liu, Yoganathan Kanagasundaram, Liang Yang, Zhao-Xun Liang

**Affiliations:** 1 School of Biological Sciences, Nanyang Technological University, Singapore, 637551 Singapore; 2 Interdisciplinary Graduate School, Nanyang Technological University, Singapore, Singapore; 3 Singapore Centre for Environmental Life Sciences Engineering (SCELSE), Nanyang Technological University, 637551 Singapore, Singapore; 4 Bioinformatics Institute, Singapore, 138671 Singapore; 5 School of Mathematics and Physics, Nanyang Technological University, Singapore, 637551 Singapore; 6 Department of Bioinformatics, School of Basic Medical Sciences, Southern Medical University, 510515 Guangzhou, China

## Abstract

*Streptomyces* are a genus of Actinobacteria capable of producing structurally diverse natural products. Here we report the isolation and characterization of a biosynthetically talented *Streptomyces* (*Streptomyces* sp. SD85) from tropical mangrove sediments. Whole-genome sequencing revealed that *Streptomyces* sp. SD85 harbors at least 52 biosynthetic gene clusters (BGCs), which constitute 21.2% of the 8.6-Mb genome. When cultivated under lab conditions, *Streptomyces* sp. SD85 produces sceliphrolactam, a 26-membered polyene macrolactam with unknown biosynthetic origin. Genome mining yielded a putative sceliphrolactam BGC (*sce*) that encodes a type I modular polyketide synthase (PKS) system, several β-amino acid starter biosynthetic enzymes, transporters, and transcriptional regulators. Using the CRISPR/Cas9–based gene knockout method, we demonstrated that the *sce* BGC is essential for sceliphrolactam biosynthesis. Unexpectedly, the PKS system encoded by *sce* is short of one module required for assembling the 26-membered macrolactam skeleton according to the collinearity rule. With experimental data disfavoring the involvement of a *trans*-PKS module, the biosynthesis of sceliphrolactam seems to be best rationalized by invoking a mechanism whereby the PKS system employs an iterative module to catalyze two successive chain extensions with different outcomes. The potential violation of the collinearity rule makes the mechanism distinct from those of other polyene macrolactams.

## Introduction

Actinobacteria are a phylum of Gram-positive bacteria renowned for the capability to produce secondary metabolites. A large number of structurally diverse natural products have been isolated from various Actinobacteria strains over the decades. Some of the natural products have become the essential part of our arsenal for treating infectious and chronic diseases. Accumulating genomic sequencing data reveals that even some of the best-studied Actinobacteria strains harbor many cryptic secondary biosynthetic gene clusters^1,2^. With more and more researchers adopting a genome-guided approach and focusing on the activation of cryptic biosynthetic gene clusters, it is expected that Actinobacteria will continue to be a rich source of novel bioactive compounds.

**Figure 1:**
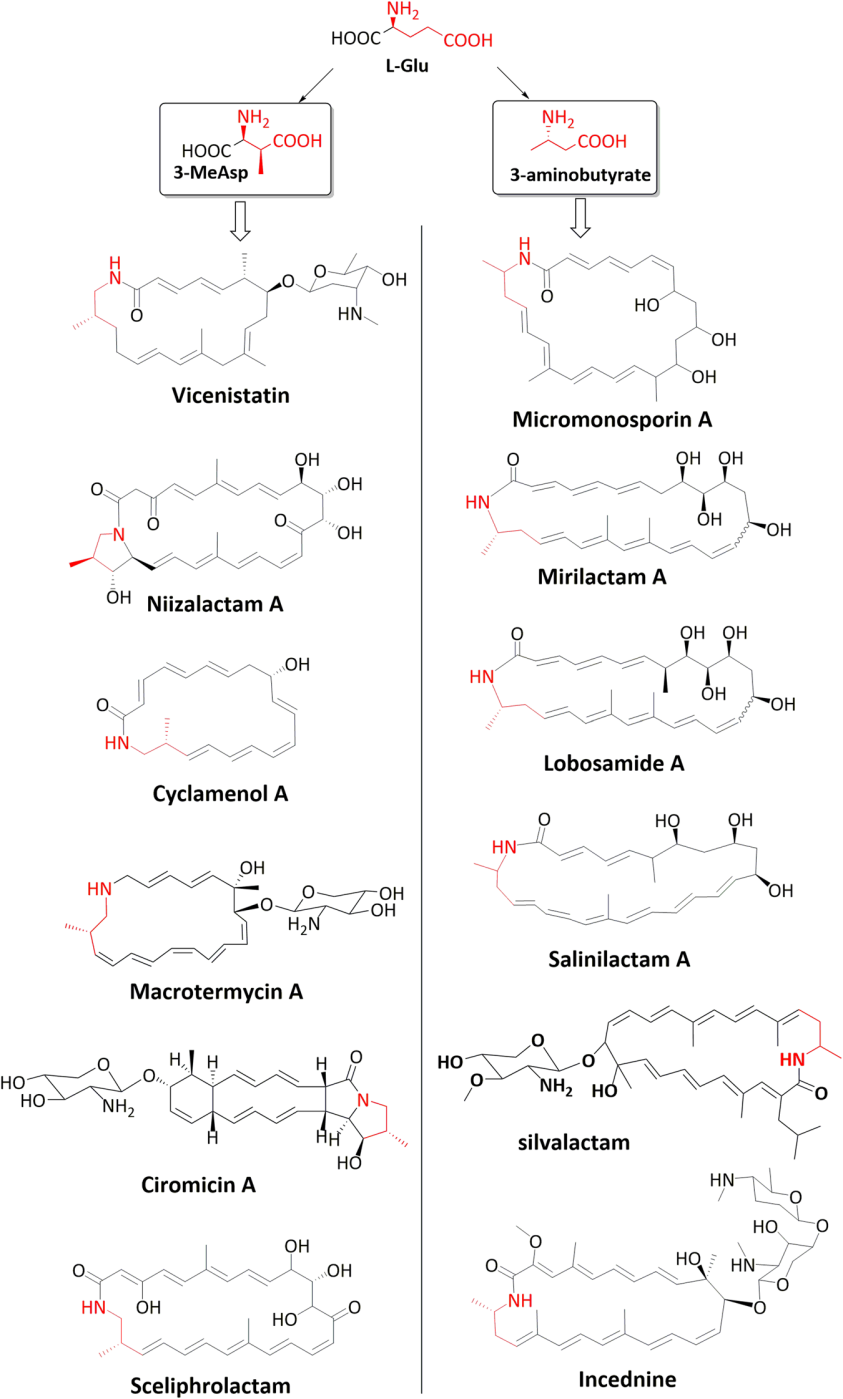
Polyene macrolactams that contain a nitrogen-containing moiety derived from L-glutamate via a β-amino acid starter unit. All the representative polyene macrolactams shown here are produced by members of the *Streptomyces* genus with the exception of micromonolactam and macrotermycin A, which are produced by *Micromonospora* and *Amycolatopsis* strains respectively.

Actinobacteria produce a superfamily of macrolactams using polyketide biosynthetic pathways. Within the macrolactam superfamily, there is a structurally distinct group of macrolactams containing a polyene skeleton and a nitrogen-containing moiety derived from L-glutamate via a β-amino acid starter unit (Fig. 1). This group of polyene macrolactams can be further divided into two subgroups according to the different β-amino acid starter units. Members of the first subgroup are synthesized using a (2S, 3S)-3-methylaspartate (3-meAsp) as starter unit^3–6^; whereas members of the second subgroup are synthesized using a 3-aminobutyrate as starter unit^7–9^. Both 3-meAsp and 3-aminobutyrate are synthesized from L-glutamate by dedicated pathways. The biosynthetic gene clusters (BGC) for vicenistatins, micromonosporin A, lobosamide A and mirlactam A have been identified and the biosynthetic mechanisms have been proposed^4,8–11^. The proposed biosynthetic mechanisms share some common features, with the macrolactam skeleton assembled by a type I modular PKS system from the L-glutamate-derived starter unit and malonyl-CoA or methylmalonyl-CoA extender units. The PKS product undergoes cyclization and tailoring to yield the final macrolactam. The so-called collinearity rule^12,13^ for polyketide biosynthesis is strictly followed in the reported biosynthetic mechanisms. Structural diversity in the polyene macrolactams is generated via the utilization of different starter units (i.e. 3-meAsp or 3-aminobutyrate), varying composition of PKS modules and different tailoring enzymes.

Here we report a biosynthetically talented *Streptomyces* strain (*Streptomyces* sp. SD85) isolated from the sediment sample collected from a coastal mangrove forest of Singapore. Among the secondary metabolites produced by *Streptomyces* sp. SD85, we isolated and identified sceliphrolactam, a polyene macrolactam that shares structural similarity and potentially similar biosynthetic mechanism with vicenistatin and some other known macrolactams. Genome sequencing and gene inactivation experiments allowed us to identify the putative sceliphrolactam BGC to reveal the enzymes involved in the synthesis of the macrolactam scaffold and starter moiety, and a potentially iterative mechanism in the biosynthesis of the macrolactam polyene scaffold.

## Results and Discussion

### *Streptomyces* sp. SD85 produces sceliphrolactam and other secondary metabolites under lab cultivation conditions

*Streptomyces* sp. SD85 is one of the Actinobacteria strains that we isolated from sediment samples collected from the mangrove forest of Sungei Buloh Wetland Reserve, Singapore. After the cultivation in GYM *Streptomyces* medium, the secondary metabolites produced by *Streptomyces* sp. SD85 were extracted from the culture broth and mycelium using ethyl acetate and methanol respectively. The secondary metabolites were subsequently profiled using liquid chromatography couple with high-resolution mass spectrometry (LC-HRMS). Aided by an in-house natural product library, we identified several known compounds that include streptorubin B (a member of prodiginine family, *m/z* = 392.2694 [M + H]^+^, calcd m/z = 392.2701 [M + H]^+^), 6,8-O-dimethylreticulol (*m/z* = 251.0916 [M + H]^+^, calcd m/z = 251.0919 [M + H]^+^), 6-O-dimethylreticulol (*m/z* = 237.0760 [M + H]^+^, calcd m/z = 237.0763 [M + H]^+^), filipin III (*m/z* = 655.4048 [M + H]^+^, calcd m/z = 655.4057 [M + H]^+^) and factumycin (*m/z* = 801.4301 [M + Na]^+^, calcd m/z = 801.4302 [M + Na]^+^). The red pigment streptorubin B is produced in abundance and accounts for the red/orange appearance of the colonies (Fig. S1). In addition, *Streptomyces* sp. SD85 produces several potentially new compounds whose spectral signatures were not found in the database. The structure for one of the “unknown” compounds was established using HRMS and NMR spectroscopy (Figs S4–S10, Table S1). This compound, which was isolated from the mycelium, turned out to be sceliphrolactam (*m/z* = 482.2532 [M + H]^+^, calcd for C28H35NO6, 482.2542 [M + H]^+^), which is a 3-amino-2-methylpropionate-containing polyene macrolactam that was isolated recently from a wasp-associated *Streptomyces* strain^11^.

### Complete genome sequencing suggests that *Streptomyces* sp. SD85 is a biosynthetically talented strain

The biosynthetic pathway for sceliphrolactam has not been elucidated as of today. To identify the sceliphrolactam BGC and to further assess the biosynthetic potential of *Streptomyces* sp. *SD85*, we first obtained a draft genome using Illumina sequencing technology. Considering that the short reads from Illumina sequencing may cause mis-assembly, we re-sequenced the genome for the second time using the single molecule real time sequencing (SMRT®) technology (PacBio) to obtain the complete genome. The complete genome, which is highly similar to the draft genome except for the gap regions, revealed that the chromosome consists of 8,625,724 base pairs with an average GC content of 72.3%. Phylogenetic analysis based on 16 S rDNA sequences suggests *S. hiroshimensis* NBRC 3839(T) as the closest relative with a shared nucleotide identity of 99.52%.

**Figure 2:**
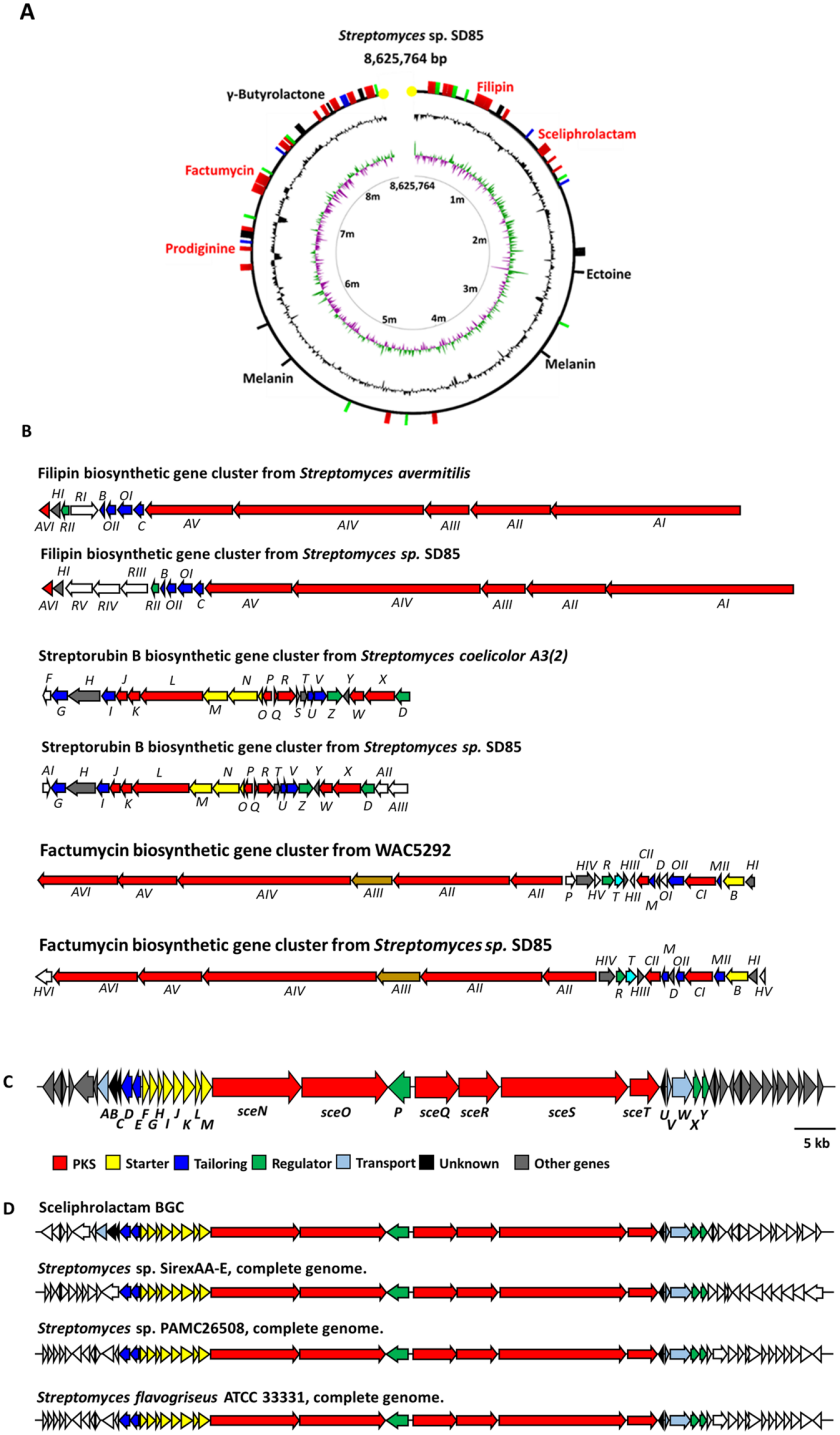
Complete genome of *Streptomyces* sp. SD85 and the PKS-based biosynthetic gene clusters (BGCs) with confirmed products. (**A**). Circular representation of *Streptomyces* sp. *SD85* chromosome. The inner ring shows a normalized GC skew plot and the center ring depicts a normalized GC content plot. The outer ring shows the distribution of secondary biosynthetic gene clusters (represented by the bars). (**B**) Comparison of the putative filipin, Streptorubin B (a prodiginine) and factumycin gene clusters from *Streptomyces* sp. SD85 with characterized homologous gene clusters from other *Streptomyces* strains. (**C**) Organization of the putative sceliphrolactam gene cluster (See Table 2 for detailed gene annotation). (**D**) Homologous gene clusters identified in three other *Streptomyces* strains.

AntiSMASH^14^ analysis using AntiSMASH 3.0 suggested that the genome contains at least 52 BGCs for the biosynthesis of different classes of secondary metabolites. The 52 BGCs occupy a total of 1.82 Mb and constitute 21.2% of the genome, which is higher than the 10.8% and 16.6% occupancy for *S. coelicolor* and *S. avermitilis*^15,16^. Similar to the *S. coelicolor* and some other *Streptomyces* strains, most of the BGCs reside in the two subtelomeric regions of the genome (Fig. 2A). More than half of the BGCs are predicted to produce polyketide and ribosome or non-ribosome peptide-derived secondary metabolites (Table 1). Seventeen BGCs have genes that encode type I, II or III PKS systems, including three type I modular PKS systems. Ten BGCs possess genes predicted to produce lantipeptide, thiopeptide or lasso-peptides; and eleven BGCs contain non-ribosomal peptide synthetase (NRPS) proteins. A few terpene BGCs were identified as evidenced by the presence of terpene synthase genes. Several BGCs are considered to be hybrid BGCs containing genes that code for more than one type of scaffold-synthesizing enzymes (Table 1). Considering that fewer than ten secondary metabolites were detected in our LC/MS-based metabolite profiling, some of the BGCs could be suppressed due to transcriptional or translational regulation.

**Table 2:** Predicted function of the genes from the sceliphrolactam BGC.

Gene	Protein Length	Closest homolog [source]	Identity/Similarity (%)	Predicted function	UniProt ID
*sceA*	456	Integral membrane transporter [*Streptomyces* sp. 769]	79/88	MFS transporter	A0A0A8EUD2
*sceB*	384	Thioredoxin [*Burkholderia cenocepacia* KC-01]	45/58	Electron transfer protein	V4ZEC6
*sceC*	73	Ferredoxin [*Saccharothrix* sp. ST-888]	61/82	Electron transfer protein	A0A0F0HKK3
*sceD*	406	Cytochrome P450 [*Streptomyces* sp. SirexAA-E / ActE]	81/87	Cytochrome P450 monooxygenase	G2NJJ2
*sceE*	404	Cytochrome P450 hydroxylase [*Streptomyces hygroscopicus subsp. jinggangensis* 5008]	81/88	Cytochrome P450 monooxygenase	H2JPQ4
*sceF*	298	Amino acid amidase [*Saccharothrix* sp. ST-888]	81/89	Peptidase	A0A0F0HK57
*sceG*	314	ACP S-malonyltransferase [Saccharothrix sp. ST-888]	79/86	Acyl carrier protein aminoacyltransferase	A0A0F0HLD6
*sceH*	87	Phosphopantetheine-binding protein [*Saccharothrix* sp. ST-888]	69/77	Acyl carrier protein	A0A0F0HNA7
*sceI*	508	ATP-dependent synthetase [*Saccharothrix* sp. ST-888]	80/86	ATP-dependent aminoacyl-ACP	A0A0F0HHV6
*sceJ*	512	Peptide synthetase [*Saccharothrix sp. ST-888*]	80/87	ATP-dependent aminoacyl-ACP	A0A0F0HNB1
*sceK*	413	Decarboxylase [*Saccharothrix* sp. ST-888]	80/89	Decarboxylase	A0A0F0HI66
*sceL*	163	Methylmalonyl-CoA mutase [*Saccharothrix* sp. ST-888]	75/86	Glutamate mutase S subunit	A0A0F0HK60
*sceM*	432	Glutamate mutase E-chain [*Streptomyces hygroscopicus subsp. jinggangensis* 5008]	75/83	Glutamate mutase E subunit	H2JPP6
*sceN*	3626	Modular polyketide synthase [*Streptomyces hygroscopicus subsp. jinggangensis 5008*]	69/76	Polyketide synthase	H2JPP5
*sceO*	3508	Modular polyketide synthase [*Streptomyces hygroscopicus subsp. jinggangensis* 5008]	74/81	Polyketide synthase	H2JPP4
*sceP*	926	LuxR family transcriptional regulator [*Streptomyces roseochromogenus subsp. oscitans* DS 12.976]	57/70	LuxR family transcriptional regulator	V6KW85
*sceQ*	1824	Polyketide synthase [*Saccharothrix* sp. ST-888]	79/85	Polyketide synthase	A0A0F0HIL7
*sceR*	1582	Beta-ketoacyl synthase [*Saccharothrix* sp. ST-888]	77/85	Polyketide synthase	A0A0F0HP09
*sceS*	5150	Modular polyketide synthase [*Streptomyces hygroscopicus subsp. jinggangensis* 5008]	73/80	Polyketide synthase	H2JPR4
*sceT*	1294	Modular polyketide synthase [*Streptomyces hygroscopicus subsp. jinggangensis* 5008]	73/81	Polyketide synthase	H2JPR3
*sceU*	81	Uncharacterized protein [*Streptomyces roseochromogenus subsp. oscitans* DS 12.976]	55/69	Unknown	V6KW03
*sceV*	266	ABC transporter [*Saccharothrix* sp. ST-888]	87/92	ABC transporter	A0A0F0HJ95
*sceW*	840	ABC transporter permease [*Streptomyces* sp. PBH53]	68/80	ABC transporter permease	A0A0H4C931
*sceX*	433	Histidine kinase [*Saccharothrix* sp. ST-888]	77/83	Histidine kinase	A0A0F0HHC4
*sceY*	214	LuxR family transcriptional regulator [*Streptomyces roseochromogenus subsp. oscitans* DS 12.976]	87/94	LuxR-family transcriptional regulator	V6KY34

**Table 1:** AntiSMASH-predicted BGCs for *Streptomyces* sp. SD85.

BGC	Position		Product/type
	From	To	
Cluster 1	128373	191397	NRPS
Cluster 2	191639	228433	Lantipeptide
Cluster 3	254893	292628	T3PKS
Cluster 4	338768	374816	Thiopeptide
Cluster 5	449647	472959	Lantipeptide
Cluster 6	544798	631900	Filipins
Cluster 7	632222	683087	NRPS
Cluster 8	752764	792909	Phosphonate
Cluster 9	814767	848264	NRPS
Cluster 10	1079383	1094356	Terpene
Cluster 11	1237686	1312223	Sceliphrolactam
Cluster 12	1368633	1383668	NRPS
Cluster 13	1460470	1474512	NRPS-T1PKS
Cluster 14	1544288	1554571	Bacteriocin
Cluster 15	1588034	1600491	Terpene
Cluster 16	1646863	1698126	NRPS-T1PKS
Cluster 17	1714938	1736137	Terpene
Cluster 18	2135476	2146348	Bacteriocin
Cluster 19	2170308	2196218	Siderophore
Cluster 20	2196485	2241910	T3 PKS-Terpene
Cluster 21	2365889	2376287	Ectoine
Cluster 22	2829683	2850624	Lantipeptide
Cluster 23	3125189	3133324	Melanin
Cluster 24	4226586	4269086	T2 PKS
Cluster 25	4476131	4497579	Lassopeptide
Cluster 26	4624864	4667988	T3 PKS
Cluster 27	4985954	5012231	Lantipeptide
Cluster 28	5634528	5639110	Melanin
Cluster 29	5999342	6017297	Siderophore
Cluster 30	6504488	6559786	NRPS
Cluster 31	6670408	6706365	Prodiginine
Cluster 32	6734497	6758278	Terpene
Cluster 33	6779444	6838577	T1 PKS-Terpene
Cluster 34	6838626	6874184	NRPS
Cluster 35	6948157	6973315	Lantipeptide
Cluster 36	7169469	7244251	PKS/FAS
Cluster 37	7248671	7339459	Factumycin
Cluster 38	7363591	7384450	Lassopeptide
Cluster 39	7568556	7585720	Terpene
Cluster 40	7611812	7663138	NRPS
Cluster 41	7695774	7723406	Lantipeptide
Cluster 42	7805055	7854201	T1 PKS-Terpene
Cluster 43	7997743	8031379	NRPS-T1 PKS
Cluster 44	8083462	8121926	NRPS
Cluster 45	8125632	8127719	γ-Butyrolactone
Cluster 46	8128024	8185901	PKS/FAS
Cluster 47	8251315	8271035	Terpene
Cluster 48	8274362	8305247	Terpene
Cluster 49	8312476	8367627	PKS/FAS
Cluster 50	8409881	8446192	Unknown
Cluster 51	8465511	8538072	PKS/FAS
Cluster 52	8549285	8564374	Lantipeptide

For PKS and NRPS proteins to be enzymatically active, the carrier protein domains of the PKS and NRPS proteins must undergo phosphopantetheinylation, a post-translational modification catalyzed by Sfp or AcpS-type 4’- phosphopantetheinyl transferases (PPTase)^17,18^. The *Streptomyces* sp. SD85 genome harbors at least three genes encoding Sfp-type PPTases and one gene-encoding AcpS-type PPTase. All four PPTases are predicted to be discrete proteins with none of them integrated into the PKS or NRPS proteins as observed for some PKSs^19^.

### Identification of sceliphrolactam BGC by gene inactivation

With the complete genome in hand, the sceliphrolactam-producing *Streptomyces* sp. SD85 strain provides us with an opportunity to identify the sceliphrolactam BGC and decipher the biosynthetic pathway. As the macrolactam skeleton of sceliphrolactam is most likely generated by a type I modular PKS system, we first searched BGCs that contained type I modular *pks* genes. The genome of *Streptomyces* sp. SD85 harbors several BGCs that are predicted to contain type I *pks* or *fas* (fatty acid synthase) genes, but only three (BGC6, 11 and 37 (Table 1)) encode type I modular PKS systems. Based on the high similarity shared with the BGCs from the MIBiG database^14^, BGC6 and BGC37 are predicted to produce filipins and kirromycin respectively (Fig. 2B)^20–22^. Assignment of the two BGCs is consistent with the detection of filipin III and factumycin (a close structural analogue of kirromycin)^23^ in the culture broth. BGC11 encodes six PKS proteins and a dozen of other enzymes, transporters and regulatory proteins (Fig. 2C). Three uncharacterized BGCs that share high similarity with BGC11 were also found in the genomes of *Streptomyces* sp. SirexAA-E*, Streptomyces* sp. PAMC26508 *and S. flavogriseus* ATCC3331 (Fig. 2D).

**Figure 3:**
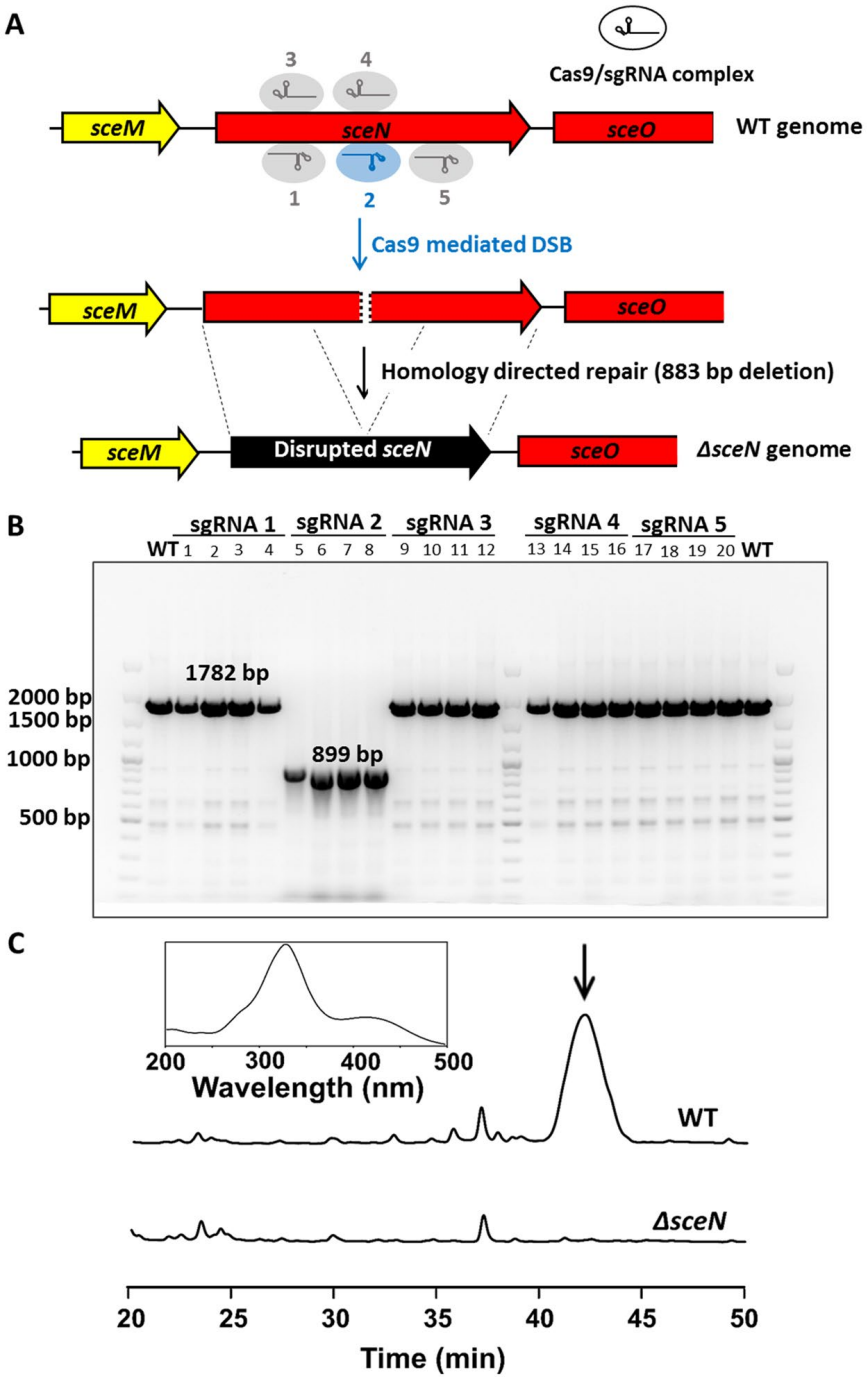
Inactivation of sceliphrolactam biosynthetic gene *sceN* using CRISPR/Cas9-based method. (**A**) Schematic illustration of the CRISPR/Cas9-mediated cleavage of genomic DNA and homology directed repair (HDR) to delete part of *sceN*. (**B**) PCR results confirmed the deletion of 883 base pairs of *sceN* using sgRNA2 as guide. A full-sized image of the DNA gel is included in the supporting information. (**C**) HPLC analysis of the *∆sceN* mutant strain to show the abolishment of sceliphrolactam production. The sceliphrolactam peak is indicated by the arrow. The wavelength (λ) was set at 330 nm for the HPLC detector (Inset: on-line absorption spectrum of sceliphrolactam).

To find out whether BGC11 is responsible for sceliphrolactam biosynthesis, we used a CRISPR/Cas9 strategy to delete an internal fragment of the *sceN* gene, which encodes the first of the six PKS modules. The pCRISPR-Cas9 plasmid was employed to express Cas9, sgRNA and a DNA template required for homology directed repair (HDR)^24^. After cloning the sgRNA and homologous DNA sequences into the pCRISPR-Cas9 plasmid, we transformed *Streptomyces* sp. SD85 with the modified plasmid by the *E. coli - Streptomyces* conjugation method^24^. After Cas9-mediated DNA cleavage and repair, DNA gel analysis and DNA sequencing confirmed that a portion of *sceN* was successfully deleted (Fig. 3A,B). It should be noted that we used five sgRNAs to target different protospacer adjacent motif (PAM) sites within the *sceN* gene and only one of the sgRNAs (sgRNA2) led to DNA cleavage and repair (Fig. 3B). As already documented by other researchers, the efficiency of Cas9/sgRNA-mediated gene deletion in bacteria can vary greatly^25^. Subsequent metabolite analysis by HPLC confirmed the absence of sceliphrolactam in the culture broth of the *∆sceN* mutant strain (Fig. 3C). The gene inactivation experiment supports that the *sce* BGC encodes the enzymes and other proteins required for sceliphrolactam biosynthesis. This conclusion is further reinforced by the sequence similarity shared by the biosynthetic genes between *sce* and the vicenistatin BGC as discussed below.

### Annotation of non-PKS biosynthetic genes in the sce gene cluster

In addition to the five PKS-encoding genes, the *sce* BGC also contains other genes predicted to code for non-PKS enzymes, membrane-embedded ABC transporters and two LuxR-type transcriptional regulators. The predicted functions of the biosynthetic enzymes, transporters and regulatory proteins are summarized in Table 1. Here we describe that the annotation of the *sce* genes provides further support for the involvement of the *sce* BGC in sceliphrolactam biosynthesis.

**Figure 4:**
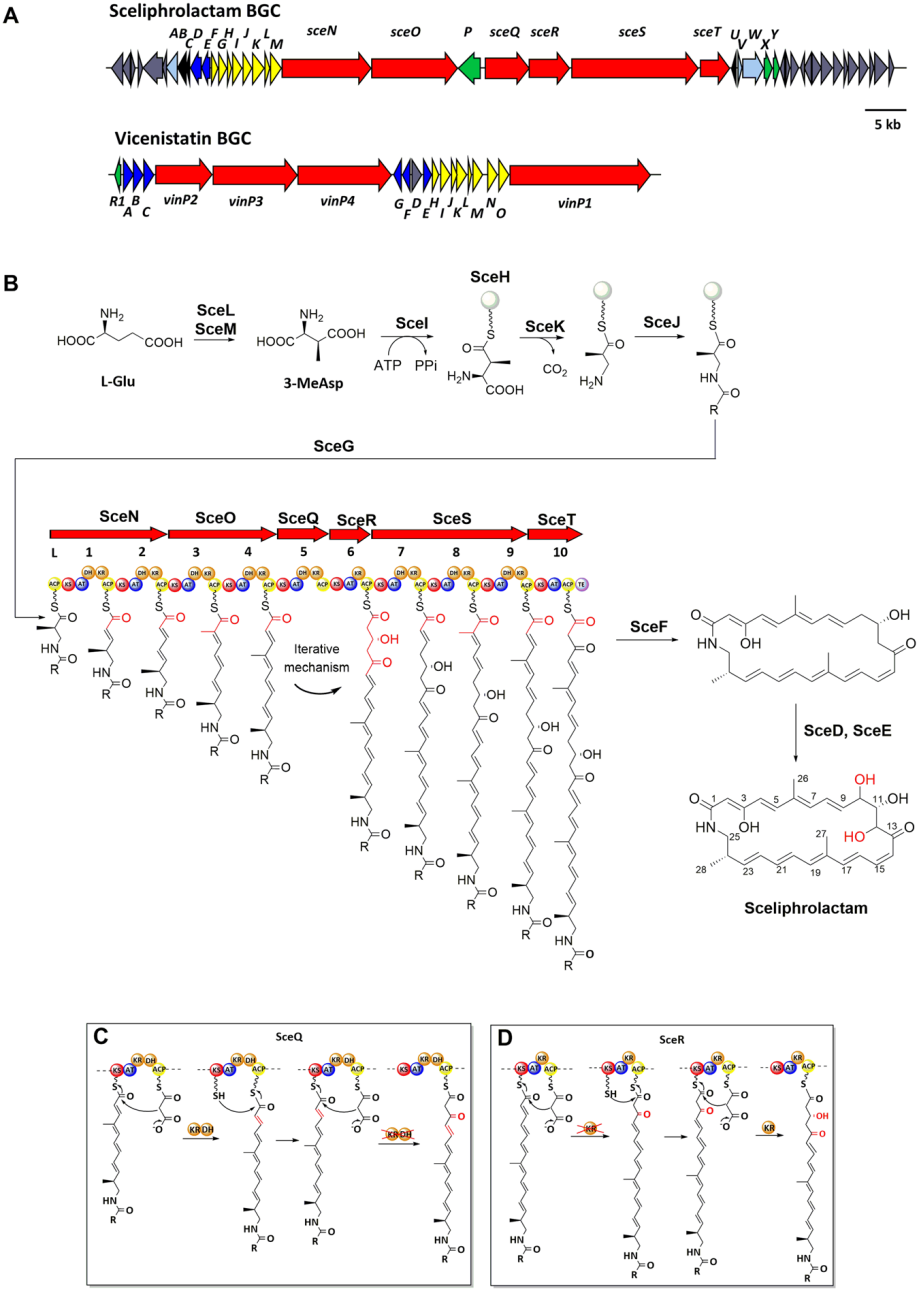
Proposed biosynthetic mechanism for sceliphrolactam. (**A**) BGCs of sceliphrolactam and vicenistatin share a set of genes (*sceG-M vs. vinH-O*) for β-amino acid starter unit biosynthesis. (**B**) Overall biosynthetic mechanism for sceliphrolactam. The presence of a *cis*-double bond between the protons of C14 and C15 was confirmed by ^1^H NOESY correlation (Fig. S10). The stereochemistry of C-11 was assigned based on the observation that the KR_6_ domain is a B-type KR domain^49–51^. (**C**) A possible mechanism with SceQ containing the iterative module. (**D**) A possible mechanism with SceR containing the iterative module. (ACP: acyl carrier protein; AT: acyltransferase; DH, dehydratase; KR; ketoreductase; KS, ketosynthase; TE, thioesterase).

The *sce* gene cluster has several genes (*sceG-N*) that share homology with a set of genes found in *S. halstedii* HC-34 for vicenistatin biosynthesis (Fig. 4A). This set of genes (*vinH-O*) is responsible for the synthesis of the 3-amino-2-methylpropionate moiety of vicenistatin from L-glutamate via 3-MeAsp^3,26^. Similar to vicenistatin, sceliphrolactam also contains a 3-amino-2-methylpropionate moiety. Considering the high sequence homology shared by the two sets of genes, the 3-amino-2-methylpropionate moiety of sceliphrolactam is likely to be synthesized by a similar pathway. Based on the function of starter-synthesizing enzymes in vicenistatin biosynthesis^27^, SceL and SceM, which are the homologs of VinH (Identity/Similarity: 57%/74%) and VinI (Identity/Similarity: 62%/70%), are likely to form a coenzyme B_12_-dependent glutamate mutase that catalyzes the transmutation of glutamate (Fig. 4B**)**. SceI, a homolog of VinN (Identity/Similarity: 61%/75%), is predicted to transfer 3-methylaspartate onto the carrier protein SceH (homolog of VinL: Identity/Similarity: 64%/75%). SceK is the homolog of VinO (Identity/Similarity: 67%/79%) and is likely to catalyze the decarboxylation and epimerization of SceH-tethered 3-methylaspartate to 3-amino-2-methyl propionate; whereas SceJ (VinM homolog, Identity/Similarity: 58%/67%) is likely to catalyze the formation of an amide to protect the reactive amino group. SceG shares high sequence homology with the aminoacyltransferase VinK (Identity/Similarity: 69%/86%), with all the catalytic residues of VinK conserved^28^. Accordingly, SceG is likely responsible for transferring the 3-amino-2-methylpropionate group from SceH onto the ACP of the loading module of SceN^26,29^.

Apart from the starter biosynthetic genes, *sceF* encodes a protein (SceF) that shares high similarity with VinJ (Identity/Similarity: 71%/84%), an amidohydrolase or peptidase in vicenistatin biosynthesis^26,30^. VinJ, which is distinct from other more common serine peptidases adopting an α/β hydrolase fold, plays a role by catalyzing the amide hydrolysis to generate the free amino group, which must be protected during the polyketide chain extension to prevent premature cyclization of intermediates^29^. Hence, the protection de-protection strategy for the reactive aminoacyl-ACP intermediates found in the biosynthesis of vicenistatin, amino marginolactone^31^ and other natural products is most likely to be shared by sceliphrolactam biosynthesis. In addition, the presence of two cytochrome P450 genes (*sceD* & *secE*) in the BGC is also consistent with the two hydroxylated carbon atoms (C-10 and C-12) in sceliphrolactam (Fig. 4B). The genes *sceA* and *sceB* encode a pair of thioredoxin and ferredoxin, which are likely to be the electron transfer proteins that supply electrons to the cytochrome P450 hydroxylases.

### Analysis of the Sce type I modular PKS

We performed detailed sequence analysis on the acyltransferase (AT), ketosynthase (KS), ketoreductase (KR) and dehydratase (DH) domains of all ten PKS modules encoded by the *sce* gene cluster. All AT, KS, KR and DH domains contain the essential catalytic residues and are predicted to be catalytically active. The substrate preference of AT domains of type-I PKS towards malonyl-CoA or methylmalonyl-CoA is defined by several key residues in the substrate binding pocket^32–34^. Sequence comparison suggests that the two AT domains from module-3 and module-8 are likely to use methylmalonyl-CoA as substrate. This is in accordance with the two methyl groups (C-26, C-27) in sceliphrolactam (Fig. 4B). Meanwhile, protein sequence analysis suggests that the KR domains belong to the “B-type” KR that contains a conserved (L/V)D(D/N) motif, which is crucial for guiding polyketide intermediates into the active site of KR domains to produce “R”-configured β-hydroxyl groups (Fig. S2).

To assemble the macrolactam skeleton of sceliphrolactam, the modular PKS system is expected to have eleven modules to join the 3-amino-2-methylpropionate starter unit with nine acetyl and two propionyl extender units. Surprisingly, the *sce* BGC only encodes ten PKS modules found in SceN, SceO, SceQ, SceR and SceS. According to the collinearity rule for PKS^12,13^, the order of PKS modules in the biosynthetic complex should be reflected in the chromosomal order of the *pks* genes. Considering the collinearity rule is strictly followed in the biosynthesis of other known macrolactam polyenes, the Sce PKS system could represent a deviation from the orthodox systems and may feature an iterative module (Fig. 4C,D) we will discuss further below. To confirm that the shortage of a module is not because of erroneous assembly of the genome sequence, DNA segments at the boundaries of s*ceO, sceQ*, *sceR* and *sceS* genes were amplified by PCR (Fig. S3). The result confirms that the four genes form a continuous DNA fragment, and thus, rules out the existence of a gene encoding a “missing” module. Meanwhile, we did not detect other macrolactams in the biomass or culture broth of *Streptomyces* sp. SD85, which makes it tenuous to argue that sceliphrolactam is a minor product generated by the aberrant stuttering of a PKS module.

### Evidence disfavoring the involvement of a trans-PKS module in sceliphrolactam biosynthesis

There is a plausible explanation for the shortage of a PKS module in the Sce system without invoking the breakdown of the collinearity rule. This would involve the use of a *trans*-PKS module encoded by another gene located somewhere else in the genome. This *trans*-PKS module must be integrated into the mega-PKS protein complex by docking specifically between SceQ and SceR. Considering that the β-keto group remains unreduced for the chain extension catalyzed by the mysterious module, a potential *trans*-PKS module is most likely to have a KS-AT-ACP domain composition and contain docking domains that are compatible with the docking domains of SceR and SceQ. However, an inspection of the complete genome of *Streptomyces* sp. SD85 revealed that all the *pks* genes in the genome are accounted for by the 52 BGCs. We could not find any orphan PKS module with docking domains, let alone an orphan module with the KS-AT-ACP domain organization. We also searched the genome of the three *Streptomyces* strains that contain the homologous *sce* BGCs (Fig. 2C) and did not find such orphan PKS module. Moreover, we identified the N- and C-terminal portions of SceN, SceO, SceQ, SceR and SceS that are predicted to fold into docking domains crucial for holding the PKS complex together (Fig. S13)^35^. The lack of a gene that encodes a potential *trans*-PKS module and the presence of compatible docking domains for SceR and SceQ seem to argue against the participation of a *trans*-PKS module.

**Figure 5:**
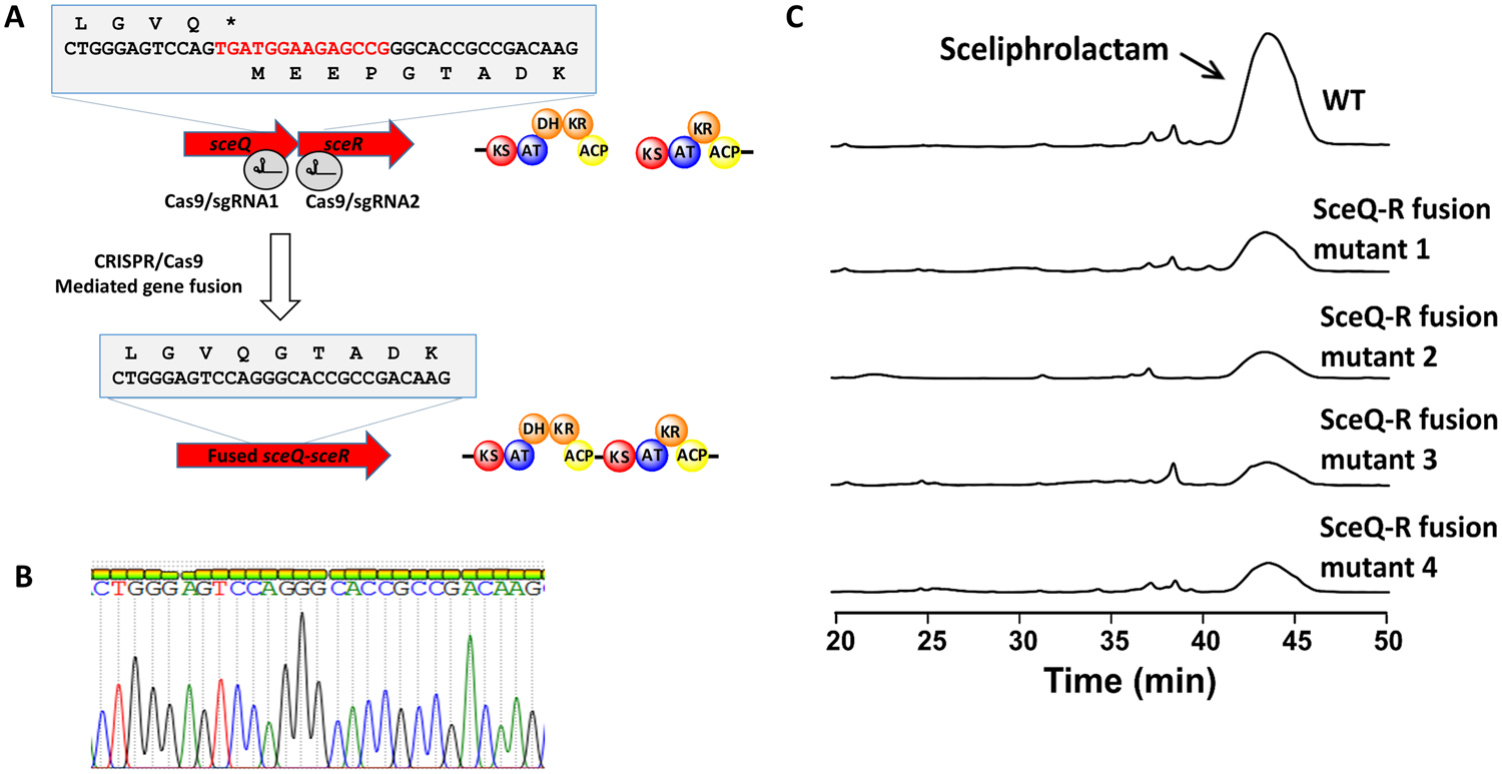
(**A**) Schematic illustration of the CRISPR/Cas9-mediated and dual sgRNA-guided DNA cleavage to fuse the *sceQ* and *sceR* genes. (**B**) DNA sequencing result confirmed the fusion of the two genes upon the deletion of 14 base pairs (sequence in red). (**C**) HPLC analysis comparing the production of sceliphrolactam between the *sceQ-R* fusion mutant and the wild type *Streptomyces* sp. SD85. The sceliphrolactam peak is indicated by the arrow. The wavelength (λ) was set at 330 nm for the HPLC detector.

To seek experimental evidence for or against the involvement of a *trans* PKS module, we constructed a mutant strain by fusing the *sceQ* and *sceR* genes on the chromosome. The rationale behind the experiment is that when the *sceQ* and *sceR* genes are fused, SceQ and SceR will be expressed as a single protein to prevent the insertion of any potential *trans*-PKS module. Production of sceliphrolactam is expected to be abolished for the fusion mutant because the *trans*-PKS module can no longer dock between the closely linked SceQ and SceR. Similar gene fusion experiment has been used to support an iterative mechanism in the biosynthesis of borrelin^36^. To create the *sceQ-R* fusion mutant, a pCRISPR-cas9 plasmid that harbors two sgRNA cassettes was constructed to target the *sceQ/sceR* intergenic region for the deletion of 14 base pairs (Fig. 5A). Deletion of the 14 base pairs was expected to remove the stop and start codons and lead to the fusion of *sceQ* and *sceR* genes. The sgRNA and homology template-containing pCRISPR-cas9 plasmid was transformed into *Streptomyces* sp. SD85 strain using the same *E. coli* - *Streptomyces* conjugation method as the gene inactivation experiment described earlier. Deletion of the 14 base pairs was confirmed by gene sequencing for the four colonies we tested (Fig. 5B). After cultivating the *sceQ-R* fusion mutant strains inoculated using four positive colonies, we found that sceliphrolactam was still produced by all four mutant strains, despite at a lower yield than the wild type strain (Fig. 5C). The results suggest that the polyketide intermediate is passed from SceQ directly to SceR, and thus, it is tenuous to argue that a *trans*-PKS module is involved in the biosynthesis of sceliphrolactam.

### A potential biosynthetic mechanism that involves an iterative PKS module

As the experimental evidence argue against the involvement of a *trans*-PKS module, the apparent lack of a PKS module in the biosynthesis of sceliphrolactam remains to be fully explained. One of the conceivable mechanisms involves the iterative use of a PKS module. The iterative use of modules in PKS systems is increasingly known as a common phenomenon and has been reported in the biosynthesis of lankacidin^37^, borrelidin^38^, aureothin^39^, stigmatellin^40^ and other polyketide secondary metabolites^41^. There are two possibilities, with either SceQ or SceR acting iteratively (Fig. 4C,D). If SceQ acts iteratively, the β-keto group is reduced in the first chain-extension cycle to generate a hydroxyl group that is subsequently dehydrated, but remains unreduced in the second cycle. On the other hand, if SceR acts iteratively, the β-keto group is not reduced in the first chain-extension cycle; but reduced to yield hydroxyl group in the second cycle. Regardless of which module acts iteratively, the iterative module seems to be “programmed” with the activity of the KR domain switching on and off in two successive extension cycles. Hence, in comparison with most known iterative mechanisms that involve modules acting iteratively with the processing domains (e.g. KR, DH, ER or MT) generating the same chemical outcome in each extension cycle, the putative iterative module in sceliphrolactam biosynthesis is able to generate different outcome during the two successive extension cycles. Such “programmed iteration” is rare but not unprecedented. It was recently found that the biosynthesis of azalomycin involves a similar iterative module. This iterative module contains an ER domain whose activity can be “toggled” off and on to ensure that the ER domain only functions in the second extension cycle^31,42^. The selective keto-reduction on different polyketide intermediates by the SceQ or SceR module is also reminiscent of the programmed keto-reduction mechanism observed in partially reducing iterative PKSs^32,43–45^.

In summary, we have isolated and characterized a biosynthetically talented *Streptomyces* strain that harbors a large number of uncharacterized biosynthetic gene clusters. As an effort to explore the biosynthetic potential of the strain, we identified all the modular PKS-based BGCs and their products. For the polyene macrolactam sceliphrolactam, identification of the *sce* BGC suggests a potentially unique modular PKS system for macrolactam biosynthesis. Our experimental data suggest that the biosynthesis of sceliphrolactam potentially involves a “programmed” iterative PKS module. Among all the known polyene macrolactams, sceliphrolactam is the only one that features a biosynthetic mechanism that does not seem to follow the co-linearity rule by involving an iterative PKS module. Future mechanistic studies are required to identify which module acts iteratively and understand how the iterative domain achieves the programmed keto-reduction.

## Experimental Procedures

### *Streptomyces* sp. SD85 isolation and maintenance

*Streptomyces* sp. SD85 was isolated from marine sediment samples collected from the Sungei Buloh Wetland Reserve, Singapore. The sediment samples were collected from the sediment (1–2 meter depth) in the mangrove swamps to harvest the microbial communities from the submerged sediment. The sediment samples were rinsed and filtered after being air dried and treated with microwave irradiation. With the filtrate, a series of 10-fold dilutions were performed and a total of 0.1 mL from each dilution was plated onto the International *Streptomyces* Project (ISP3) medium plate. 30 μg/mL Cycloheximide and 25 μg/mL nalidixic acid were used to inhibit fungal and fast growing Gram-negative bacteria. All culture plates were incubated for up to three weeks. Individual colonies were obtained after a few rounds of streaking and the strain was maintained in GYM medium agar (4 g/L glucose, 4 g/L yeast extract, 10 g/L malt extract, 20 g/L agar, pH 7.2) at 28 °C.

### Complete genome sequencing

High quality genomic DNA was extracted using a modified genomic DNA extraction protocol for *Streptomyces*^46^. The genomic DNA was first sequenced using illumine technology to obtain a draft genome which contains large gaps. Then the complete genome sequence of *Streptomyces* sp. SD85 was obtained using Single Molecule Real Time (SMRT) sequencing technology (Pacific Biosciences, California, USA). We sequenced the genome on a PacBio RS II platform. 1 SMRT cell was used and the sequencing run was performed 3 times. The PacBio long sequencing reads have reached an approximate 114 X coverage of the genome, and were successfully assembled into a linear chromosome with a size of 8,625,764 bp by using the Hierarchical Genome Assembly Process 2 (HGAP2) protocol from SMRT Analysis version 2.0. The DNA sequences for the sceliphrolactam biosynthetic gene cluster obtained from illumina and PacBio sequencing are identical and have been deposited in GenBank (Accession number: KX230849). For Illumina MiSeq sequencing, a total of 3,906,106 reads were obtained with an average read length of 145.17 bases. 3,896,491 reads were matched with an average length of 145.34 bases. Sequencing reads were trimmed and *De novo* assembly was performed using CLC Genomics Workbench (CLC bio, Denmark). A total of 112 contigs were obtained with an average length of 76,406 bases and a total length of 8,557,547 bases. For the long-read sequencing performed the PacBio technology, a single SMRT cell was used with a three sequencing runs. A read quality value of 84 was achieved with 86,939 zero-mode-waveguides (ZMWs), with average coverage value of 114.0. Mean polymerase read length was 15,610 bp with mean reads of inserts (ROI) readlength value of 13,164 bp. A single contig of length 8,625,764 bases was obtained after assembly.

### Profiling of secondary metabolites in the culture broth and mycelia of Streptomyces sp. SD85

*Streptomyces* sp. 85 was cultured in GYM liquid medium (100 mL in 500 mL baffled Erlenmeyer flask) for 96 hr at 28 °C. Mycelia were separated from culture broth by centrifugation. Mycelia and culture broth were extracted separately with methanol and ethyl acetate, respectively. The organic layers were dried under vacuum and re-dissolved in methanol. HPLC analysis of the crude extract was performed with Grace® VisionHT^TM^ C18-HL (4.6 mm × 250 mm, 5 µm) using an Agilent 1200 HPLC system equipped with DAD detector. A 60 min gradient elution at 1 mL/min was employed with 10% aqueous acetonitrile to 100% acetonitrile. Mobile phase solvents were supplemented with 0.1% formic acid. Analytes were monitored at λ = 220 nm, 280 nm, 330 nm, 400 nm. LC-MS was performed using Thermo Scientific Accela LC system coupled with an LTQ XL^TM^ Linear Ion Trap Mass Spectrometer. The conditions for LC were similar to those employed for HPLC-DAD analysis. Mass spectra were measured using positive and negative ion mode of ESI (capillary voltage 3.5 kV; cone voltages 30 V/50 V; source temperature 400 °C; cone gas flow 60 L/hr; desolvation gas flow 450 L/hr) with a mass range of 100–2000 Da and a scan time of 0.5 s.

### Large-scale fermentation and isolation of sceliphrolactam

*Streptomyces* sp. SD85 was cultured in GYM liquid medium (800 mL in 2 L baffled Erlenmeyer flask) for 7 days at 29 °C, in the dark. Combined mycelia from four culture flasks were extracted with one liter of acetone twice. The organic layer was dried under vacuum to afford a crude extract (~4 g). The extract was re-dissolved in 90% methanol and partitioned four times with equal volume of hexane to remove unwanted lipid material. The washed extract was subjected to semi-preparative reversed phase HPLC using Shimadzu Prominence Preparative HPLC system equipped with Zorbax Eclipse XDB C18 semi-prep column (9.4 mm × 250 mm, 5 µm). An isocratic solvent system of H_2_O:CH_3_OH (45:55) with a flow rate of 4.7 mL/min was used for compound isolation. Elution of the compounds was monitored at 333 nm and a peak corresponding to sceliphrolactam was observed at t = 22 min. Pure sceliphrolactam (2 mg/L) was obtained under dark conditions to minimize degradation of the polyene compound.

### Construction of *Streptomyces* sp. SD85 ∆sceN mutants using CRISPR/Cas9 method

Construction of the plasmid pCRISPR-Cas9-SceN for CRISPR/Cas9-mediated *sceN* gene mutation in *Streptomyces* sp. *SD85* was performed using pCRISPR-Cas9, which was a kind gift from Sang Yup Lee’s lab in the Korea Advanced Institute of Science and Technology (KAIST). All cloning steps were carried out using *E. coli* TOP10 (Invitrogen, US). Identification of protospacer sequences in *sceN* was performed using the Benchling Server. Selected *sceN* DNA spacer was introduced into pCRISPR-Cas9 by ligating a PCR-generated sgRNA sequence into *Sna*BI and *Nco*I linearized pCRISPR-Cas9 vector. To generate an 883 bp deletion via homology directed repair, two DNA templates that are homologous to the 5′- and 3′- regions of the *sceN* gene and are 883 bp apart from each other were amplified from genomic DNA using Phusion® high fidelity DNA polymerase. The sceN spacer-containing pCRISPR-Cas9 was digested using *Stu*I restriction enzyme and jointed with the template DNA fragments using Gibson Assembly kit (New England BioLabs, US). The sgRNA and template-containing pCRISPR-Cas9 plasmid was introduced into the *Streptomyces* sp. SD85 parental strain by conjugation method using *E. coli* ET12567/pUZ8002 as described^47^. Briefly, *Streptomyces* spores were collected from seven-day old culture grown on GYM agar containing 10 mM MgCl_2_ and 10 mM CaCl_2_, and subjected to heat shock at 45 °C for 5 min. They were allowed to germinate at 30 °C for 6 hr and mixed with an exponential phase culture of *E. coli* ET12567/pUZ8002 containing the modified pCRISPR-Cas9 plasmid at a donor to recipient ratio of 3:1. The resultant mixture was plated onto MS agar supplemented with 40 mM MgCl_2_ and 20 mM CaCl_2_, and incubated for 22.5 hr at 30 °C. One millilitre of sterile water containing 1 mg of apramycin and 500 µg of nalidixic acid was overlaid onto the plates for selection of successful exconjugants. Plates were further incubated at 30 °C for 4–5 days before exconjugants were picked onto fresh MS agar plates containing 40 μg/mL apramycin and 25 µg/mL nalidixic acid.

Exconjugants harbouring the modified pCRISPR-Cas9 plasmid were determined by colony PCR. True exconjugants were patched onto MS agar that contain 40 μg/mL apramycin and 10 μg/mL thiostrepton for induction of Cas9 expression. Clearance of plasmids was achieved by patching resultant colonies onto MS agar and incubating them at 37 °C for 4 days. Apramycin sensitivity was confirmed by replica plating onto selective and non-selective plates.

To screen for successful mutants, individual clones were cultured in liquid GYM at 30 °C for 3 days for genomic DNA isolation using a previously described protocol^48^. Diagnostic PCR was performed to establish a deletion of 883 bp in the *sceN* gene.

### CRISPR/Cas9 mediated sceQ-R gene fusion

A dual sgRNA-containing pCRISPR-Cas9 plasmid was constructed by designing a dual sgRNA synthetic construct with a configuration of (*Nco*I site – spacer - sgRNA1tracr – terminator -*gapdhp* (EL) - spacer - sgRNAtracr – *Sna*BI site). This construct was cloned into *Sna*BI and *Nco*I linearized pCRISPR-Cas9 vector by Gibson Assembly. The resulted plasmid was linearized using the *Stu*I restriction enzyme and ligated with the HDR template to yield the plasmid pCRISPR-cas9-dual-sceQR. Introduction of this plasmid into *Streptomyces sp. SD85* and subsequent induction of Cas9 expression was performed as described above. Successful mutant strains were validated by diagnostic PCR and sequencing of the fragments amplified from genomic DNA of individual clones.

### HPLC analysis of sceliphrolactam production for the *Streptomyces* sp. SD85 ∆sceN and sceQ-R fusion mutants

The mycelia of 7 days old wild-type (WT) *Streptomyces* sp. SD85 and the mutant strains were extracted twice with equal volume of acetone and dried under vacuum. The crude extracts were re-dissolved in methanol and subjected to HPLC analysis with an Agilent 1200 HPLC system equipped with DAD for UV detection. A Gradient elution program was employed, starting with 40% aqueous methanol at 0 min to 70% aqueous methanol at 50 min and 100% methanol at 60 min. The analytes were monitored at λ = 220 nm, 280 nm, 330 nm, 420 nm. Experiments were repeated twice to ensure reproducibility.

### Accession code

The BGC gene sequence has been deposited in public database (GenBank accession number: KX230849).

## Electronic supplementary material

Supporting Information
