# Supplementary material for: Identification of a biosynthetic gene cluster for the polyene macrolactam sceliphrolactam in a Streptomyces strain isolated from mangrove sediment
Source: Sci Rep. 2018 Jan 25;8:1594. doi: 10.1038/s41598-018-20018-8 (PMC5785472; doi:10.1038/s41598-018-20018-8)
Supplement: Supplementary file 1 — Supporting Information [file 41598_2018_20018_MOESM1_ESM.pdf]

Identification of a biosynthetic gene cluster for the polyene macrolactam sceliphrolactam in a *Streptomyces* strain isolated from mangrove sediment

Zhen Jie Low,† Li Mei Pang,† Yichen Ding,† Qing Wei Cheang,† Kim Le Mai Hoang,⊥ Hoa Thi Tran,† Jinming Li,# Xue-Wei Liu,⊥ Yoganathan Kanagasundaram,ǂ Liang Yang,† Zhao-Xun Liang*,†

† School of Biological Sciences, Nanyang Technological University, Singapore 637551
ǂ Bioinformatics Institute, Singapore 138671
⊥ School of Mathematics and Physics, Nanyang Technological University, Singapore 637551
# Department of Bioinformatics, School of Basic Medical Sciences, Southern Medical University, 510515 Guangzhou, China.

Electronic supplementary information

Table S1. ^{1}H and ^{13}C NMR data for sceliphrolactam (DMSO-d_6, 500MHz)..2

Table S2. Primers and sequences used in this study 3

Figure S1. Growth of *Streptomyces sp.* SD85 on solid medium. 4

Figure S2. Prediction of stereochemistry of β-hydroxyl group and olefin structures through KR fingerprint. 4

Figure S3. Verification of sequence boundaries between *sceP* to *sceS*.. 5

Figure S4. ^{1}H NMR spectrum of sceliphrolactam in DMSO-d_6, 500 MHz. 6

Figure S5. ^{13}C NMR spectrum of sceliphrolactam in DMSO-d_6, 500 MHz. 6

Figure S6. COSY spectrum of sceliphrolactam in DMSO-d_6, 500 MHz. 7

Figure S7. TOCSY spectrum of sceliphrolactam in DMSO-d_6, 500 MHz. 7

Figure S8. HSQC spectrum of sceliphrolactam in DMSO-d_6, 500 MHz. 8

Figure S9. HMBC spectrum of sceliphrolactam in DMSO-d_6, 500 MHz. 8

Figure S10. 1D ^{1}H NOESY spectrum of sceliphrolactam in DMSO-d_6, 500 MHz 9

Figure S11. Mass spectrum of sceliphrolactam. 9

Figure S12. Full-sized DNA agarose gel image showing the results of CRISPR gene knockout. The image for the central part of the gel is shown as Fig 2b. 10

Figure S13. Analysis of docking domains and effect of *sceR-Q* gene fusion on sceliphrolactam biosynthesis 10

Table S1. ^{1}H and ^{13}C NMR data for sceliphrolactam (DMSO-d_6, 500 MHz)

no.	δ_H	δ_C	Type
1		172.4	C
2	4.99 s	93.7	CH
3		165.6	C
3-OH	13.68 s		
4	5.84 d (15.0)	121.6	CH
5	6.53 d (15.0)	138.2	CH
6		134.5	C
7	5.85 d (13.5)	135.8	CH
8	6.36 dd (13.5, 12.0)	(128.3)[a]	CH
9	5.42 dd (13.5, 7.5)	136.7	CH
10	4.04 dd (9.0, 7.5)	70.1	CH
10-OH	4.78 br.s		
11	3.79 br.d (9.0)	(74.5)[a]	CH
11-OH	4.78 br.s		
12	4.25 br.s	79.6	CH
12-OH	4.99 s		
13		(201.7)[b]	C
14	6.21 m	119.9	CH
15	6.63 m	146.5	CH
16	7.39 t (14.0)	125.5	CH
17	6.67 m	143.5	CH
18		135.3	C
19	6.24 m	135.9	CH
20	6.21 m	135.7	CH
21	6.19 m	127.4	CH
22	5.81 dd (15.0, 11.0)	131.4	CH
23	5.36 dd (15.0, 9.0)	138.1	CH
24	2.20 m	(39.3)[a]	CH
25a	3.04 m	(43.9)[a]	CH
25b	2.97 m		
25-NH	7.74 s		
26	1.80 s	12.0	CH3
27	1.60 s	12.0	CH3
28	1.00 d (6.0)	16.2	CH3

[a]From HSQC correlation
[b] From HBMC correlation

Table S2. Primers and sequences used in this study.

Primer name	Sequence (5' -> 3')	Purpose
Ctg1_20 sgRNA F	CATGCCATGGgtcgccggatatcaccacggGTTTTAGAGCTAGAAATAGC	sgRNA amplifcation
Ctg1_20 sgRNA2 F	CATGCCATGGcccgtcgccggatatcaccaGTTTTAGAGCTAGAAATAGC	
Ctg1_20 sgRNA3 F	CATGCCATGGcccgcctccgtggtgatatcGTTTTAGAGCTAGAAATAGC	
Ctg1_20 sgRNA4 F	CATGCCATGGccgtggtgatatccggcgacGTTTTAGAGCTAGAAATAGC	
Ctg1_20 sgRNA5 F	CATGCCATGGcgcggacgtgttcgacccagGTTTTAGAGCTAGAAATAGC	
sgRNA R	ACGCCTACGTAAAAAAAGCACCGACTCGGTGCC	
sgRNA check F	CACAGGAAACAGCTATGACC	sgRNA sequencing
sgRNA check R2	TCAGCCCAGATCCCCGATCC	
sgRNA check R	ACCCCCCATTCAAGAACAGC	
SceN left F	TCGTCGAAGGCACTAGAAGGTCGATGCCTACCTGCTGACC	Homologous recombination template construction for pCRISPR-Cas9-SceN
SceN left R	TTGCGCTGGAAGCGGTAGGTTGGAGCTGCATGTCGAGGTA	
SceN right F	ACCTACCGCTTCCAGCGCAA	
SceN right R	GGTCGATCCCCGCATATAGGGCGAAGTCACCGTCGTGGT	
pcrispr-cas9 seq	TGAGGCTTGCAGGGAGTCAA	HDR template sequencing
pcrispr-cas9 seq R	CGTCGCTCTCTGGCAAAGCT	
SceN check F	GCTCGGCTCGGTGAAGTCGA	Validation of sceN deletion
SceN check R	AAGAGCACGCGGCCGGAGAT	
Boundary 1F	TTCCAGTTCTTTATGGCGGTC	Boundary between sceP and sceQ
Boundary 1R	GAAGAAGTCGTGGTCGAACT	
Boundary 2F	CAGGAAATGGGCTTCGACTC	Boundary between sceQ and sceR
Boundary 2R	GAGACGATGCTGCCGGATAT	
Boundary 3F	TTCGTGCTCTTCTCCTCCCTC	Boundary between sceR and sceS
Boundary 3R	GATGCCCAGGGTGTACGAGAT	
SceQ-R left F40	TCGTCGAAGGCACTAGAAGGTGGAACCTGCACGAACTCAC	Homologous recombination template construction for pCRISPR-Cas9-SceQ-R
SceQ-R left R40	CGGAGCTTGTCGGCGGTGCCCTGGACTCCCAGTTCGTTGT	
SceQ-R right F20	GGCACCGCCGACAAGCTCCG	
SceQ-R right R40	GGTCGATCCCCGCATATAGGGATCGAGCCGAGCCACATGG	
SceQ-R check F2	AAATGGGCTTCGACTCGCTC	PCR screening of sceQ-R fusion mutant
SceQ-R check R2	GAAGCCGGACACCACGTCGA	
SceQ-R check F2	AAATGGGCTTCGACTCGCTC	Sequencing of PCR amplified fragment of sceQ-R fusion mutant
SceQ-R check R2	GAAGCCGGACACCACGTCGA	

Sequence name	Sequence (5' -> 3')	Purpose
SceQ-R spacer 1	gagtccagtgatggaagagc	protospacer sequence
SceQ-R spacer 2	agtccagtgatggaagagcc	
gapdhp(EL) promoter	GCTGCTCCTTCGGTCGGACGTGCGTCTACGGGCACCTTACCGCAGCC GTCGGCTGTGCGACACGGACGGATCGGGCGAACTGGCCGATGCTG GGAGAAGCGCGCTGCTGTACGGCGCGCACCGGGTGCGGAGCCCCT CGGCGAGCGGTGTGAAACTTCTGTGAATGGCCTGTTCGGTTGCTTTT TTTATACGGCTGCCAGATAAGGCTTGCAGCATCTGGGCGGCTACCG CTATGATCGGGGCGTTCCTGCAATTCTTAGTGCGAGTATCTGAAAG GGGATACGC	Promoter sequence for dual sgRNA synthetic construct
oop terminator	ACTCCATCTGGATTTGTTCAGAACGCTCGGTTGCCGCCGGGCGTTTT TTA	terminator sequence for dual sgRNA synthetic construct

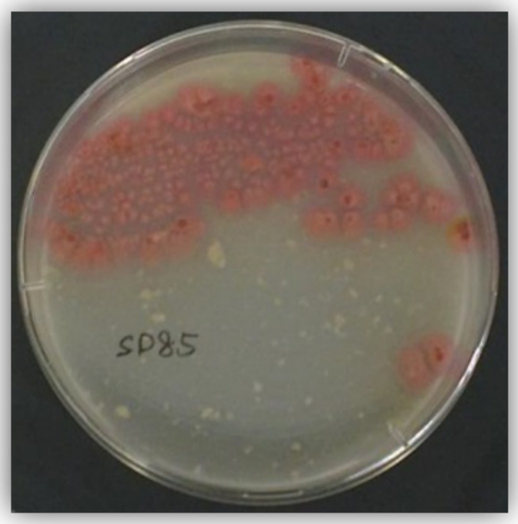

Figure S1. Growth of *Streptomyces sp.* SD85 on solid medium. *Streptomyces* sp. SD85 was inoculated on a modified ISP 2 medium (4 g/L glucose, 4 g/L yeast extract, 1 g/L malt extract and 12 g/L of bacto agar) and incubated at 28 °C for 7 days.

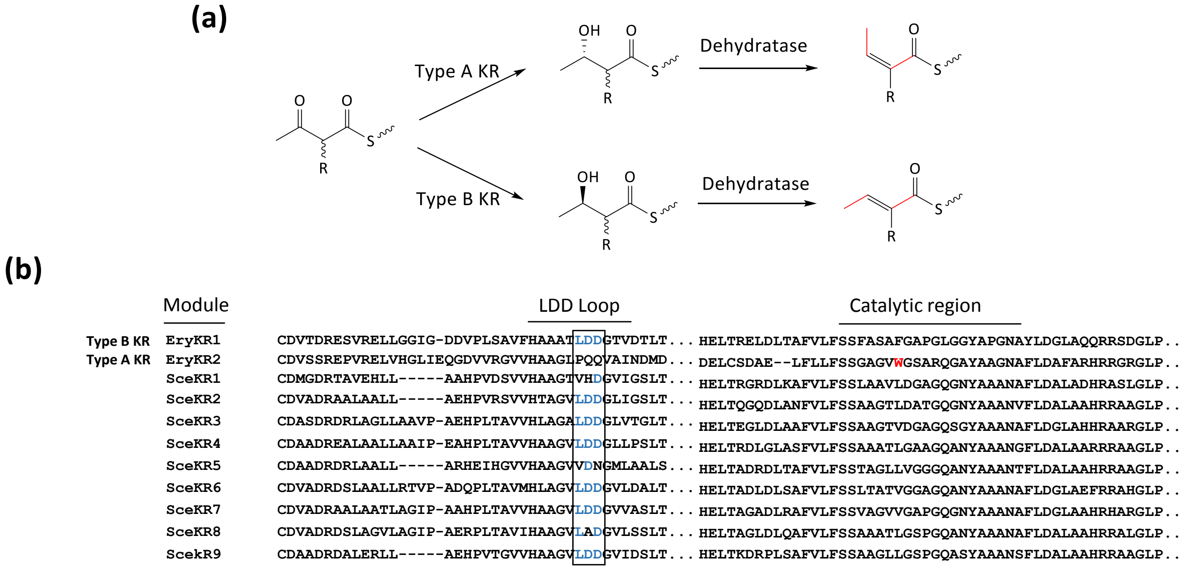

Figure S2. Prediction of stereochemistry of β-hydroxyl group and olefin structures through KR fingerprint. (a) Geometry of olefin structure from dehydration by DH is dependent on chirality of β-hydroxyl group generated by upstream KR domain. (b) Comparison of KR fingerprint from Sce PKS modules with A-Type and B-Type KR examples obtained from erythromycin PKS.

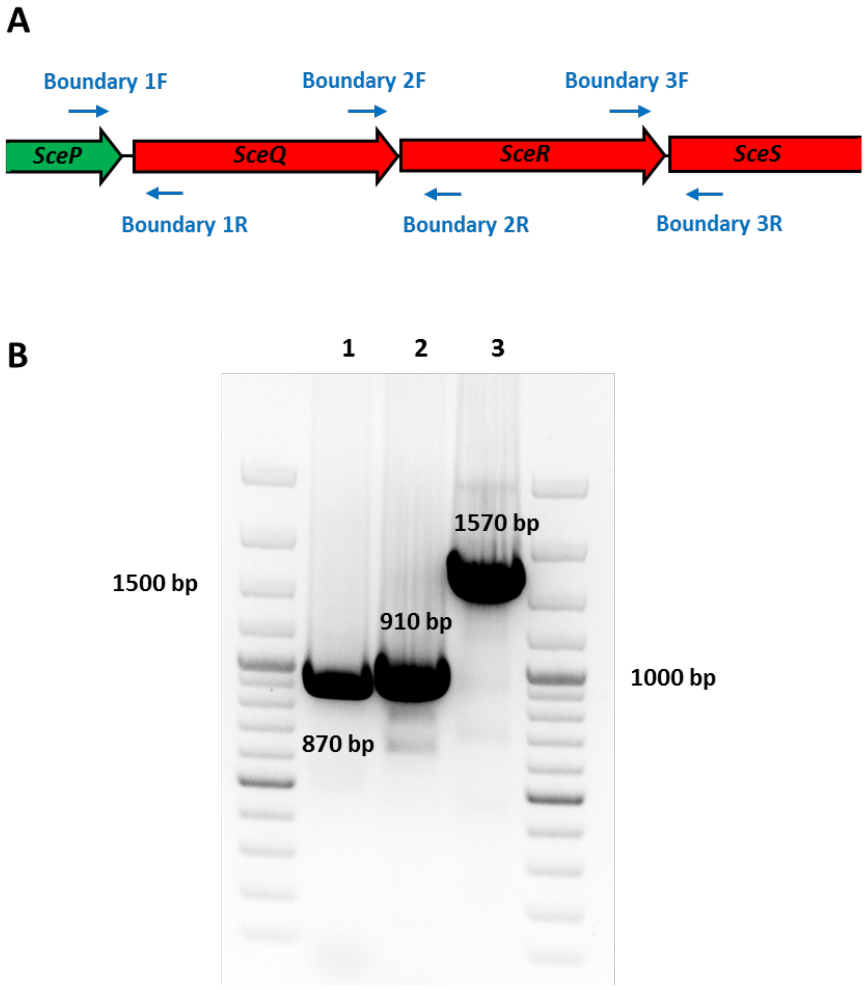

Figure S3. Verification of sequence boundaries between *sceP* to *sceS*. (A) Three sets of primers were designed to amplify the sequence between *sceP* to *sceQ*, *sceQ* to *sceR* and *sceR* to *sceS*. (B) Band sizes of PCR products were in accordance to predicted DNA sizes based on *sce* gene cluster sequence. Sequences of the PCR products were confirmed via DNA sequencing.

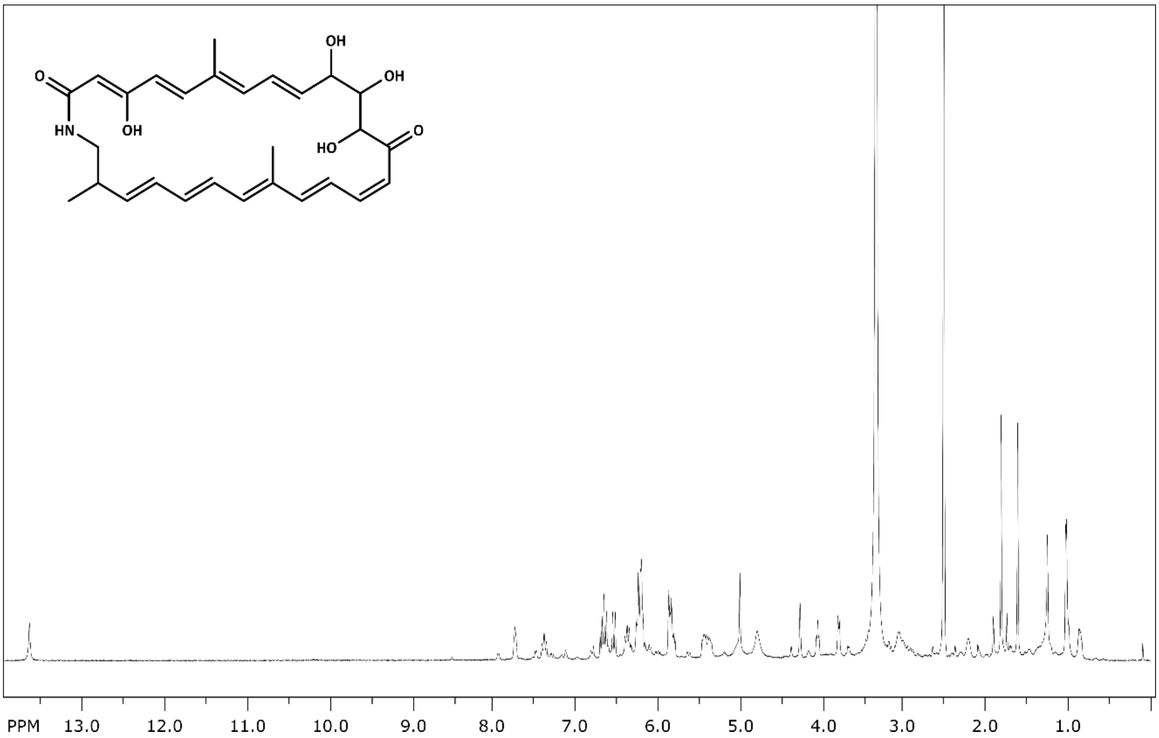

Figure S4. ^{1}H NMR spectrum of sceliphrolactam in DMSO-d_6, 500 MHz.

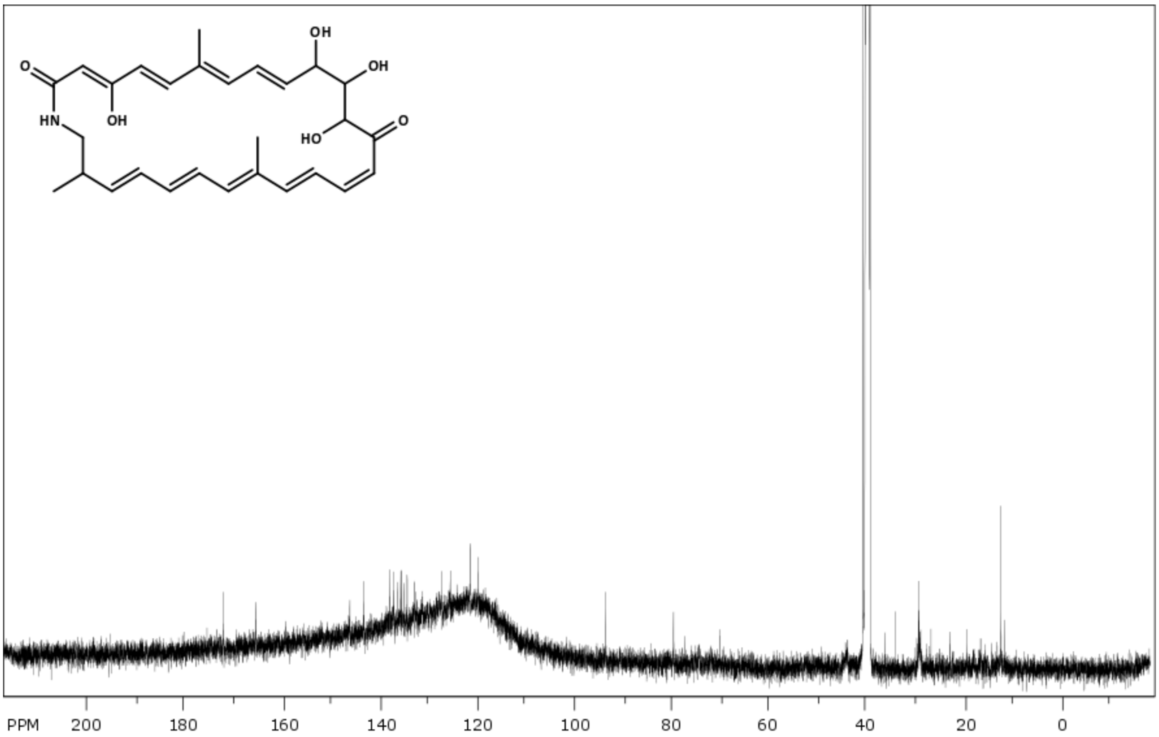

Figure S5. ^{13}C NMR spectrum of sceliphrolactam in DMSO-d_6, 500 MHz.

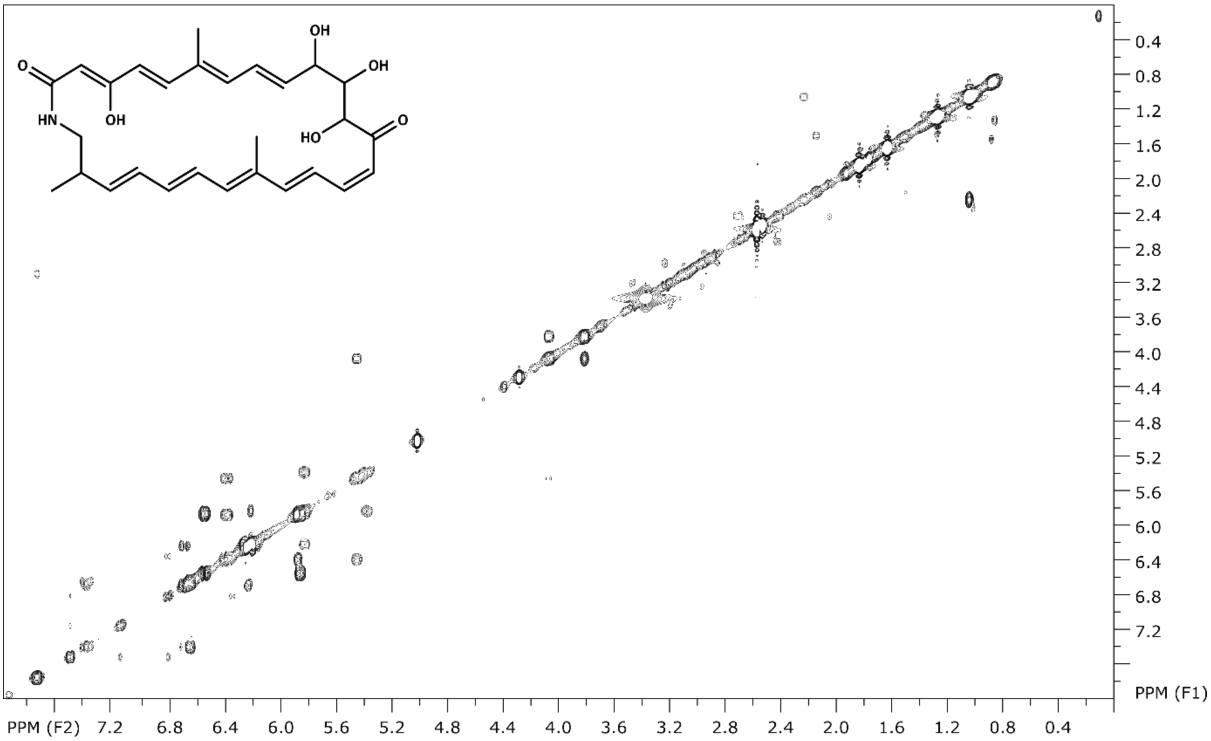

Figure S6. COSY spectrum of sceliphrolactam in DMSO-d_6, 500 MHz.

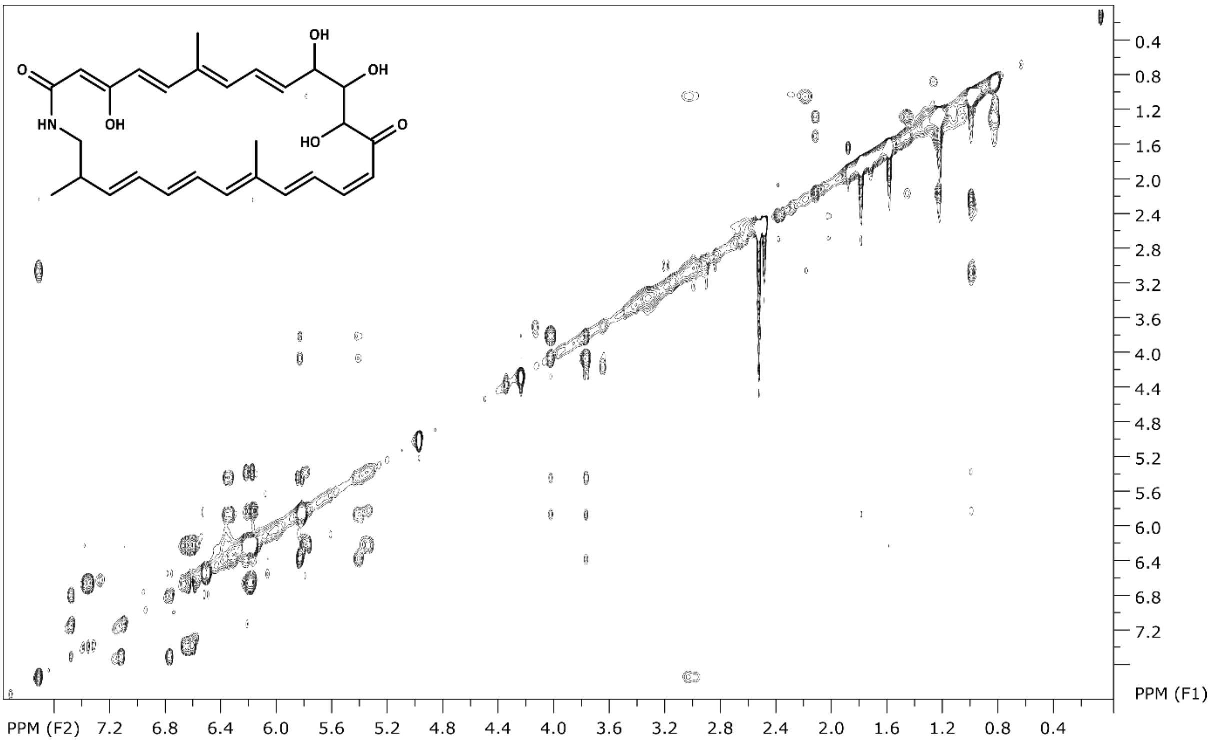

Figure S7. TOCSY spectrum of sceliphrolactam in DMSO-d_6, 500 MHz.

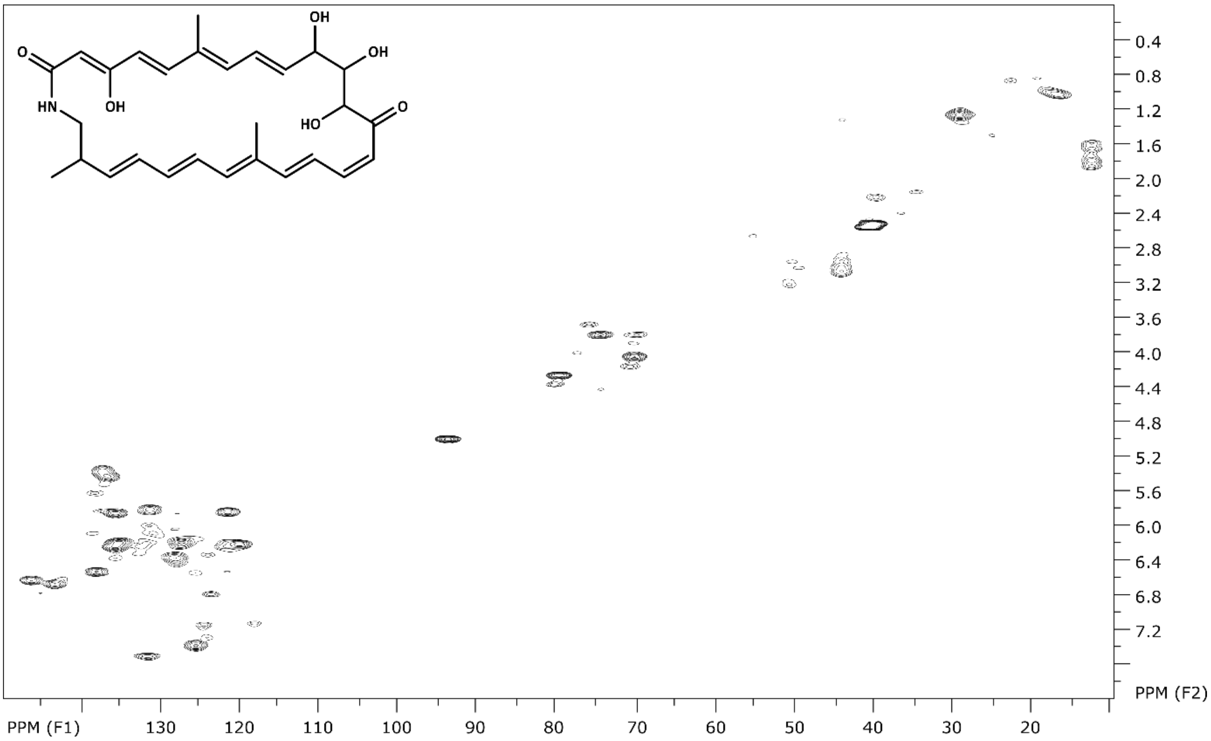

Figure S8. HSQC spectrum of sceliphrolactam in DMSO-d_6, 500 MHz.

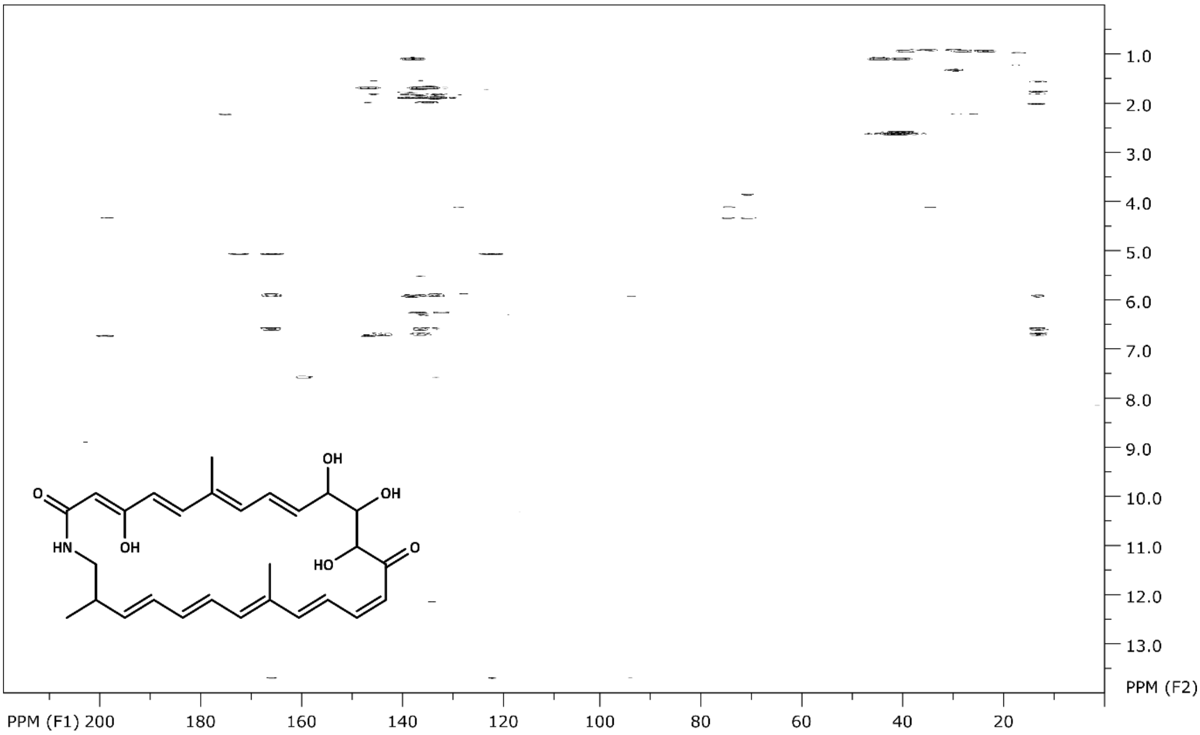

Figure S9. HMBC spectrum of sceliphrolactam in DMSO-d_6, 500 MHz.

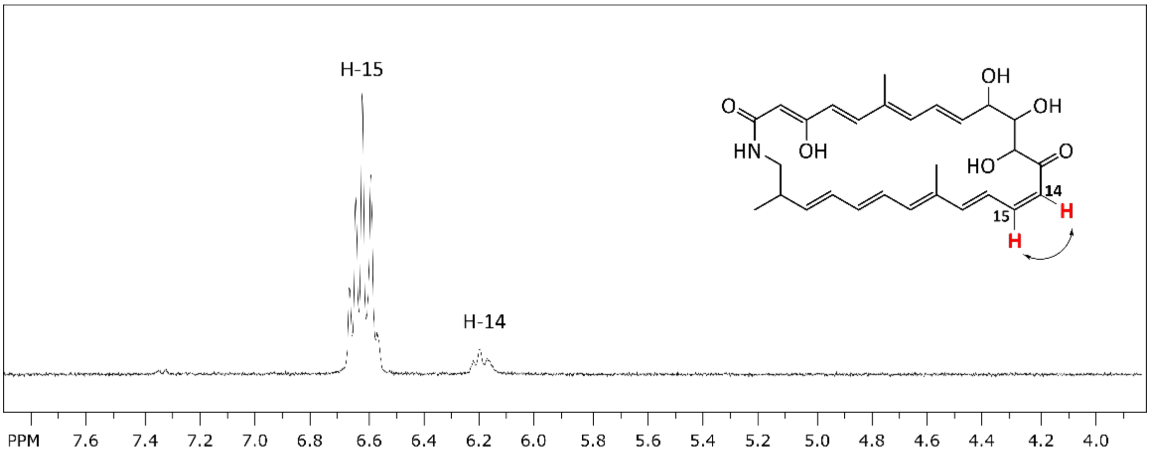

Figure S10. 1D ^{1}H NOESY spectrum of sceliphrolactam (DMSO-d_6, 500 MHz) with selective excitation of H-15 to establish the geometry of olefin connecting H-15 and H-14.

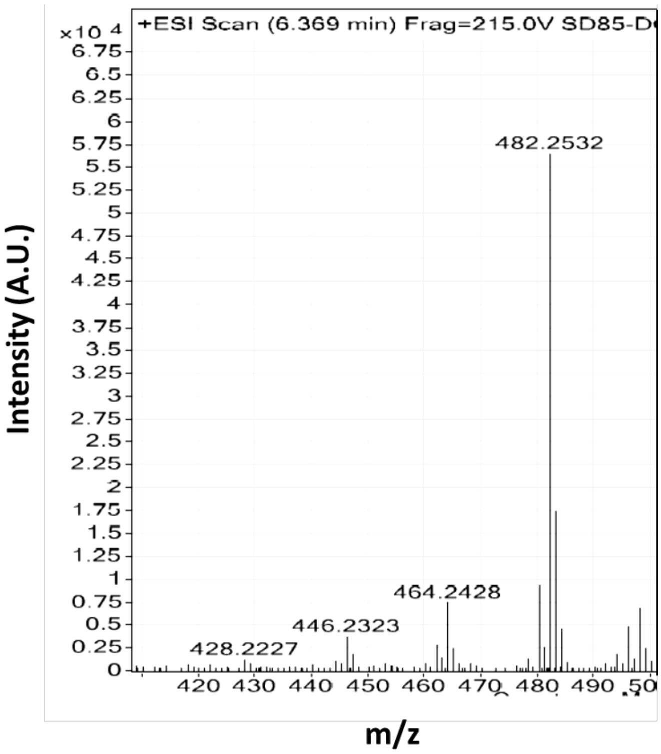

Figure S11. Mass spectrum of sceliphrolactam. The data was collected using Agilent G6540 QTOF (+ESI scan) Frag = 215.0V.

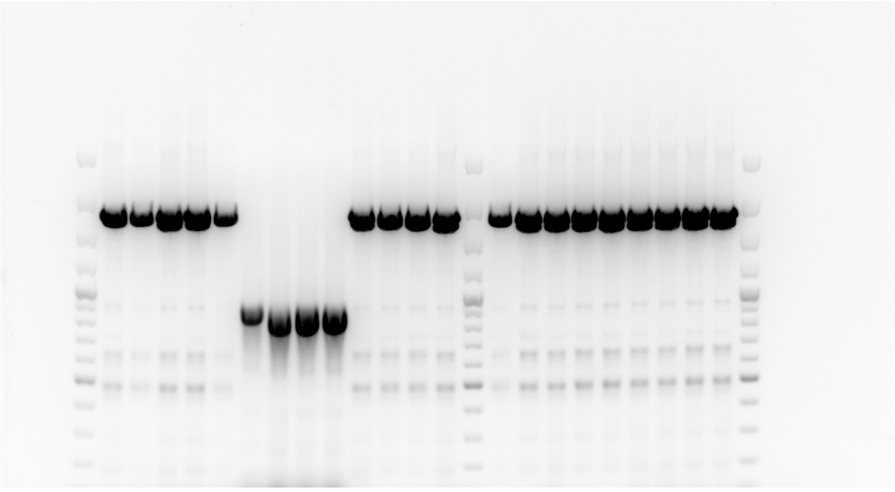

Figure S12. Full-sized DNA agarose gel image showing the results of CRISPR gene knockout. The image for the central part of the gel is shown as Fig 2b.

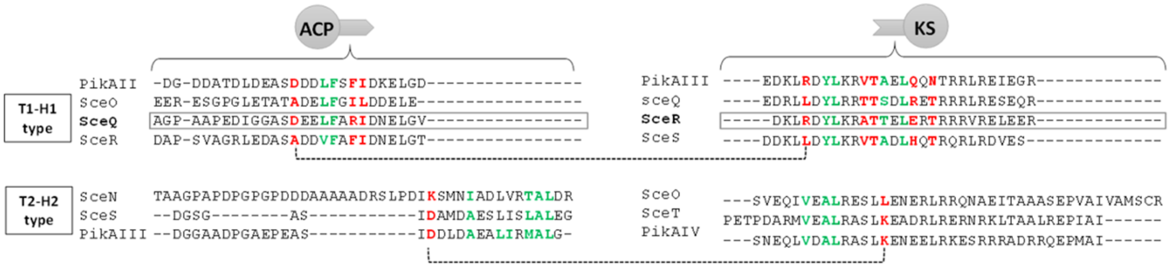

Figure S13. Analysis of docking domains and effect of *sceR-Q* gene fusion on sceliphrolactam biosynthesis. Multiple sequence alignment of the docking domains of Sce PKS system. Each row shows an interacting head (H) – tail (T) pair, with the key residues involved in specific domain-domain interaction highlighted in red and green according to previous studies [1,2]. The two residues connected by dash lines link are matching pairs that engage in charge-charge or hydrophobic interaction. The compatible H1-T1 type docking domain pair for sceQ-SceR are shown in the box. According to the classification of PKS docking domains [1,2], the paired docking domains for SceO/SceQ, SceQ/SceR, SceR/SceS belong to the H1-T1 type; whereas the docking domains for SceN/SceO and SceS/SceT belong to the H2-T2 type. The docking domains of SceQ and SceR share high sequence similarity with those of the PikAII/PikAIII pair from pikromycin biosynthesis [2,3], with the matching Asp/Arg pair and non-polar residues (Fig. 5A). The compatibility of the docking domains suggests that SceQ and SceR are able to interact with each other through the docking domains.

References

1 Thattai, M., Burak, Y. & Shraiman, B. I. The origins of specificity in polyketide synthase protein interactions. *PLoS Comput. Biol.* **3**, 1827-1835 (2007).

2 Buchholz, T. J. *et al.* Structural Basis for Binding Specificity between Subclasses of Modular Polyketide Synthase Docking Domains. *ACS Chem. Biol.* **4**, 41-52 (2009).

3 Chen, S., Xue, Y. Q., Sherman, D. H. & Reynolds, K. A. Mechanisms of molecular recognition in the pikromycin polyketide synthase. *Chem. Biol.* **7**, 907-918 (2000).
